# *N*-[Eth­yl(2-hy­droxy­eth­yl)carbamo­thio­yl]-3-fluoro­benzamide

**DOI:** 10.1107/S1600536814008174

**Published:** 2014-04-18

**Authors:** Nor Wahida. Awang, Siti Aishah Hasbullah, Siti Fairus M. Yusoff, Bohari M. Yamin

**Affiliations:** a School of Chemical Sciences and Food Technology, Universiti Kebangsaan Malaysia, 43600 Bangi, Selangor, Malaysia; b Low Carbon Research Group, School of Chemical Sciences and Food Technology, Universiti Kebangsaan Malaysia, 43600 Bangi, Selangor, Malaysia

## Abstract

In the title compound, C_12_H_15_FN_2_O_2_S, the mol­ecule adopts a *cis* configuration of the fluoro­benzoyl group with respect to the thiono group about their C—N bond. The dihedral angle between the fluoro­benzoyl group and the thio­urea N_2_CS fragment is 69.60 (11)°. An intra­molecular N—H⋯O hydrogen bond occurs. In the crystal, mol­ecules form chains along the *b*-axis direction *via* O—H⋯S and C—H⋯O hydrogen bonds.

## Related literature   

For bond length data see: Allen *et al.* (1987 ▶). For a related structure, see: Yamin *et al.* (2014 ▶).
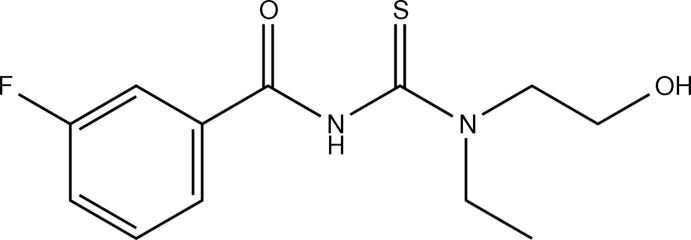


## Experimental   

### 

#### Crystal data  

C_12_H_15_FN_2_O_2_S
*M* *_r_* = 270.32
Orthorhombic, 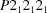

*a* = 6.0205 (3) Å
*b* = 12.9441 (6) Å
*c* = 17.1071 (9) Å
*V* = 1333.16 (11) Å^3^
*Z* = 4
Mo *K*α radiation
μ = 0.25 mm^−1^
*T* = 296 K
0.50 × 0.50 × 0.29 mm

#### Data collection  

Bruker SMART APEX CCD area-detector diffractometer
Absorption correction: multi-scan (*SADABS*; Bruker, 2000 ▶) *T*_min_ = 0.885, *T*_max_ = 0.931
21708 measured reflections
3290 independent reflections
2864 reflections with *I* > 2σ(*I*)
*R*_int_ = 0.031

#### Refinement  

*R*[*F* ^2^ > 2σ(*F* ^2^)] = 0.036
*wR*(*F* ^2^) = 0.086
*S* = 1.07
3290 reflections
168 parameters
1 restraint
H atoms treated by a mixture of independent and constrained refinement
Δρ_max_ = 0.22 e Å^−3^
Δρ_min_ = −0.18 e Å^−3^
Absolute structure: Flack (1983 ▶), 1378 Friedel pairs
Absolute structure parameter: −0.05 (8)

### 

Data collection: *SMART* (Bruker, 2000 ▶); cell refinement: *SAINT* (Bruker, 2000 ▶); data reduction: *SAINT*; program(s) used to solve structure: *SHELXS97* (Sheldrick, 2008 ▶); program(s) used to refine structure: *SHELXL97* (Sheldrick, 2008 ▶); molecular graphics: *SHELXTL* (Sheldrick, 2008 ▶); software used to prepare material for publication: *SHELXTL* and *PLATON* (Spek, 2009 ▶).

## Supplementary Material

Crystal structure: contains datablock(s) global, I. DOI: 10.1107/S1600536814008174/bq2395sup1.cif

Structure factors: contains datablock(s) I. DOI: 10.1107/S1600536814008174/bq2395Isup2.hkl

Supporting information file. DOI: 10.1107/S1600536814008174/bq2395Isup3.cml

CCDC reference: 996752

Additional supporting information:  crystallographic information; 3D view; checkCIF report

## Figures and Tables

**Table 1:** Hydrogen-bond geometry (Å, °)

*D*—H⋯*A*	*D*—H	H⋯*A*	*D*⋯*A*	*D*—H⋯*A*
N1—H1*A*⋯O2	0.86	2.03	2.805 (2)	150
O2—H2*A*⋯S1^i^	0.82 (3)	2.49 (3)	3.2805 (19)	166 (3)
C11—H11*B*⋯O1^ii^	0.97	2.44	3.259 (3)	142

Symmetry codes: (i) 
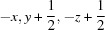
; (ii) 
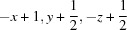
.

**Fig. 1.**
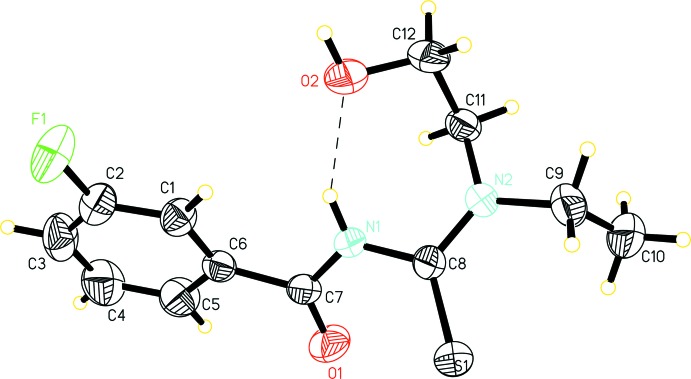
: Molecular structure of (I) with 50% probability displacement ellipsoids. The dashed line indicates intramolecular hydrogen bonds.

**Fig. 2.**
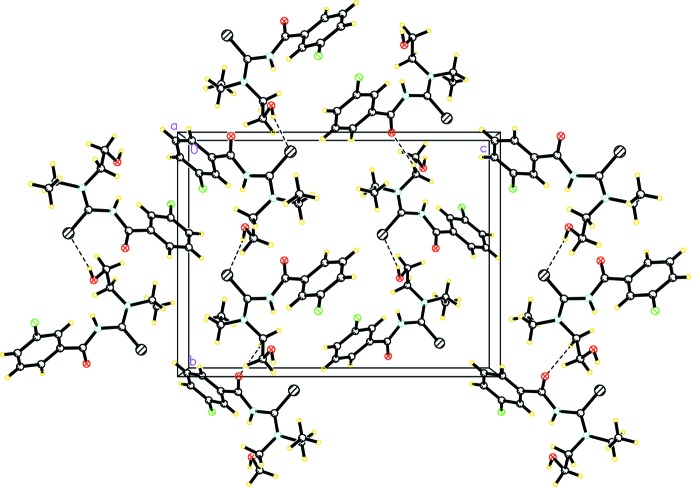
: Molecular packing (I) viewed down a axis. The dashed lines indicate intramolecular hydrogen bonds.

## supplementary crystallographic information

### 1. Comment

The expected *cis* configuration of the carbonoyl with respect to the thione group was observed when one terminal of the thiourea moiety is a secondary amine as in the case of 2,4-dichloro-*N*-[ethyl(2-hydroxyethyl)- carbamothioyl]benzamide (Yamin *et al.*, 2014). Such configuration will enhance its property as bidentate ligand in a complexation reaction with metals. The title compound is similar but having a monosubstituted flourine atom at position-3 of the benzene ring (Fig 1). The fluorobenzoyl group is also *cis* to the thiono group, C8—S1 about the N1—C8 bond. The thiourea moiety S1/N1/N2/C8 and fluorobenzene ring F1/(C1—C6) are planar with maximum deviation of 0.022 (2) Å for C8 atom from the least square plane of the thiourea moiety fragment. The two planes make dihedral angle of 69.60 (11)°, slightly less than that of the analog (75.41 (8)°). The bond lengths and angles are in normal ranges (Allen *et al.*, 1987). There is intramolecular hydrogen bond between hydroxyl oxygen atom, (O2) and the hydrogen of the amide group. In the crystal structure, molecules are linked by O2—H2A···S1 and C11—H11B···O1 intermolecular hydrogen bonds (see Table 1 for symmetry codes) to form one-dimensional chain along the *b* axis (Fig.2).

### 2. Experimental

A mixture of acetone (30 ml) solution and 2-(ethylamino)ethanol (0.18 g, 2 mmol) was added into round-bottom flask containing 4-fluorobenzoyl isothiocyanate (0.36 g, 2 mmol). The mixture was refluxed for 3 h. The mixture then cooled and filtered off. The filtrate was left to evaporate at room temperature. The solid formed was washed with water and cold ethanol. Crystals suitable for X-ray study were obtained by recrystallization from ethanol.

### 3. Refinement

After their location in the difference map, the H-atoms attached to the C and N atoms were fixed geometrically at ideal positions and allowed to ride on the parent atoms with C—H = 0.93 Å, with *U*_iso_(H)=1.2*U*_eq_(C). The hydrogen atom attached to oxygen atom was located from Fourier map and refined isotropically with O—H restraint to 0.82 with an e.s.d. of 0.01. The rotating model was applied for the refinement of methyl H atoms.

### Crystal data

**Table:**

C_12_H_15_FN_2_O_2_S	*F*(000) = 568
*M**_r_* = 270.32	*D*_x_ = 1.347 Mg m^−^^3^
Orthorhombic, *P*2_1_2_1_2_1_	Mo *K*α radiation, λ = 0.71073 Å
Hall symbol: P 2ac 2ab	Cell parameters from 11689 reflections
*a* = 6.0205 (3) Å	θ = 3.1–28.2°
*b* = 12.9441 (6) Å	µ = 0.25 mm^−^^1^
*c* = 17.1071 (9) Å	*T* = 296 K
*V* = 1333.16 (11) Å^3^	Block, colorless
*Z* = 4	0.50 × 0.50 × 0.29 mm

### Data collection

**Table:**

Bruker SMART APEX CCD area-detector diffractometer	3290 independent reflections
Radiation source: fine-focus sealed tube	2864 reflections with *I* > 2σ(*I*)
Graphite monochromator	*R*_int_ = 0.031
ω scans	θ_max_ = 28.2°, θ_min_ = 3.1°
Absorption correction: multi-scan (*SADABS*; Bruker, 2000)	*h* = −8→7
*T*_min_ = 0.885, *T*_max_ = 0.931	*k* = −17→16
21708 measured reflections	*l* = −21→22

### Refinement

**Table:**

Refinement on *F*^2^	Secondary atom site location: difference Fourier map
Least-squares matrix: full	Hydrogen site location: inferred from neighbouring sites
*R*[*F*^2^ > 2σ(*F*^2^)] = 0.036	H atoms treated by a mixture of independent and constrained refinement
*wR*(*F*^2^) = 0.086	*w* = 1/[σ^2^(*F*_o_^2^) + (0.0367*P*)^2^ + 0.3143*P*] where *P* = (*F*_o_^2^ + 2*F*_c_^2^)/3
*S* = 1.07	(Δ/σ)_max_ < 0.001
3290 reflections	Δρ_max_ = 0.22 e Å^−^^3^
168 parameters	Δρ_min_ = −0.18 e Å^−^^3^
1 restraint	Absolute structure: Flack (1983), 1378 Friedel pairs
Primary atom site location: structure-invariant direct methods	Absolute structure parameter: −0.05 (8)

### Special details

**Table:**

Geometry. All e.s.d.'s (except the e.s.d. in the dihedral angle between two l.s. planes) are estimated using the full covariance matrix. The cell e.s.d.'s are taken into account individually in the estimation of e.s.d.'s in distances, angles and torsion angles; correlations between e.s.d.'s in cell parameters are only used when they are defined by crystal symmetry. An approximate (isotropic) treatment of cell e.s.d.'s is used for estimating e.s.d.'s involving l.s. planes.
Refinement. Refinement of *F*^2^ against ALL reflections. The weighted *R*-factor *wR* and goodness of fit *S* are based on *F*^2^, conventional *R*-factors *R* are based on *F*, with *F* set to zero for negative *F*^2^. The threshold expression of *F*^2^ > σ(*F*^2^) is used only for calculating *R*-factors(gt) *etc*. and is not relevant to the choice of reflections for refinement. *R*-factors based on *F*^2^ are statistically about twice as large as those based on *F*, and *R*- factors based on ALL data will be even larger.

### Fractional atomic coordinates and isotropic or equivalent isotropic displacement parameters (Å^2^)

**Table:**

	*x*	*y*	*z*	*U*_iso_*/*U*_eq_	
F1	−0.5492 (2)	0.22048 (12)	0.06124 (8)	0.0690 (4)	
S1	0.25957 (10)	0.08756 (4)	0.35274 (3)	0.05288 (15)	
O1	0.2778 (3)	0.02641 (9)	0.17285 (8)	0.0537 (4)	
O2	0.0320 (2)	0.39036 (10)	0.21034 (9)	0.0501 (3)	
H2A	−0.053 (3)	0.4382 (14)	0.2024 (14)	0.067 (7)*	
N1	0.1564 (2)	0.18174 (10)	0.21762 (8)	0.0369 (3)	
H1A	0.0836	0.2359	0.2039	0.044*	
N2	0.3727 (2)	0.27479 (10)	0.30261 (8)	0.0360 (3)	
C1	−0.2089 (3)	0.16654 (13)	0.11163 (9)	0.0382 (4)	
H1	−0.2228	0.2134	0.1526	0.046*	
C2	−0.3693 (3)	0.15905 (16)	0.05543 (10)	0.0438 (4)	
C3	−0.3574 (4)	0.09202 (19)	−0.00610 (11)	0.0556 (5)	
H3	−0.4698	0.0890	−0.0433	0.067*	
C4	−0.1751 (4)	0.02952 (18)	−0.01123 (13)	0.0625 (6)	
H4	−0.1633	−0.0167	−0.0526	0.075*	
C5	−0.0084 (4)	0.03385 (15)	0.04385 (11)	0.0497 (5)	
H5	0.1148	−0.0091	0.0394	0.060*	
C6	−0.0245 (3)	0.10242 (12)	0.10612 (10)	0.0341 (3)	
C7	0.1516 (3)	0.09866 (12)	0.16784 (10)	0.0349 (3)	
C8	0.2701 (3)	0.18551 (11)	0.28899 (10)	0.0348 (3)	
C9	0.4712 (4)	0.29815 (16)	0.37932 (11)	0.0546 (5)	
H9A	0.3884	0.2621	0.4196	0.066*	
H9B	0.4587	0.3717	0.3893	0.066*	
C10	0.7126 (4)	0.26688 (19)	0.38404 (16)	0.0765 (8)	
H10A	0.7259	0.1941	0.3743	0.115*	
H10B	0.7690	0.2823	0.4352	0.115*	
H10C	0.7964	0.3044	0.3456	0.115*	
C11	0.4091 (3)	0.35433 (13)	0.24232 (10)	0.0366 (4)	
H11A	0.4176	0.3211	0.1916	0.044*	
H11B	0.5505	0.3879	0.2520	0.044*	
C12	0.2301 (4)	0.43491 (12)	0.24008 (11)	0.0447 (4)	
H12A	0.2041	0.4615	0.2923	0.054*	
H12B	0.2760	0.4919	0.2070	0.054*	

### Atomic displacement parameters (Å^2^)

**Table:**

	*U*^11^	*U*^22^	*U*^33^	*U*^12^	*U*^13^	*U*^23^
F1	0.0511 (7)	0.0961 (10)	0.0598 (7)	0.0225 (7)	−0.0128 (6)	−0.0004 (7)
S1	0.0634 (3)	0.0408 (2)	0.0544 (3)	−0.0138 (2)	−0.0199 (3)	0.0157 (2)
O1	0.0596 (9)	0.0393 (6)	0.0622 (8)	0.0182 (7)	−0.0127 (7)	−0.0037 (6)
O2	0.0487 (8)	0.0337 (7)	0.0677 (9)	0.0113 (6)	−0.0071 (7)	−0.0017 (6)
N1	0.0404 (8)	0.0252 (6)	0.0451 (8)	0.0027 (6)	−0.0149 (6)	0.0016 (6)
N2	0.0388 (8)	0.0307 (7)	0.0385 (7)	−0.0034 (6)	−0.0073 (6)	−0.0005 (6)
C1	0.0421 (10)	0.0422 (8)	0.0301 (7)	0.0018 (8)	−0.0015 (7)	−0.0009 (6)
C2	0.0367 (9)	0.0559 (11)	0.0389 (9)	0.0023 (8)	−0.0004 (8)	0.0067 (8)
C3	0.0562 (12)	0.0745 (14)	0.0360 (9)	−0.0134 (12)	−0.0118 (9)	0.0010 (10)
C4	0.0816 (17)	0.0612 (13)	0.0446 (11)	−0.0042 (13)	−0.0055 (11)	−0.0176 (10)
C5	0.0578 (12)	0.0452 (10)	0.0460 (11)	0.0046 (9)	−0.0001 (9)	−0.0088 (9)
C6	0.0377 (8)	0.0304 (8)	0.0343 (8)	−0.0033 (7)	−0.0008 (7)	0.0019 (6)
C7	0.0355 (8)	0.0278 (7)	0.0413 (9)	−0.0010 (7)	−0.0014 (7)	0.0033 (6)
C8	0.0304 (8)	0.0315 (7)	0.0427 (8)	0.0003 (7)	−0.0065 (7)	0.0005 (6)
C9	0.0727 (14)	0.0475 (11)	0.0437 (10)	−0.0147 (10)	−0.0170 (10)	−0.0025 (9)
C10	0.0724 (17)	0.0650 (14)	0.0920 (17)	−0.0156 (13)	−0.0453 (15)	0.0178 (13)
C11	0.0368 (9)	0.0304 (8)	0.0425 (9)	−0.0040 (7)	−0.0002 (7)	0.0001 (7)
C12	0.0554 (12)	0.0275 (7)	0.0512 (10)	0.0047 (9)	−0.0025 (10)	−0.0039 (7)

### Geometric parameters (Å, º)

**Table:**

F1—C2	1.347 (2)	C4—C5	1.377 (3)
S1—C8	1.6736 (16)	C4—H4	0.9300
O1—C7	1.208 (2)	C5—C6	1.390 (2)
O2—C12	1.419 (2)	C5—H5	0.9300
O2—H2A	0.816 (10)	C6—C7	1.497 (2)
N1—C7	1.372 (2)	C9—C10	1.511 (3)
N1—C8	1.401 (2)	C9—H9A	0.9700
N1—H1A	0.8600	C9—H9B	0.9700
N2—C8	1.331 (2)	C10—H10A	0.9600
N2—C9	1.471 (2)	C10—H10B	0.9600
N2—C11	1.474 (2)	C10—H10C	0.9600
C1—C2	1.366 (2)	C11—C12	1.500 (3)
C1—C6	1.389 (2)	C11—H11A	0.9700
C1—H1	0.9300	C11—H11B	0.9700
C2—C3	1.366 (3)	C12—H12A	0.9700
C3—C4	1.366 (3)	C12—H12B	0.9700
C3—H3	0.9300		
			
C12—O2—H2A	106.3 (18)	N2—C8—N1	114.19 (13)
C7—N1—C8	125.36 (13)	N2—C8—S1	124.15 (13)
C7—N1—H1A	117.3	N1—C8—S1	121.54 (12)
C8—N1—H1A	117.3	N2—C9—C10	112.35 (19)
C8—N2—C9	121.44 (15)	N2—C9—H9A	109.1
C8—N2—C11	123.58 (14)	C10—C9—H9A	109.1
C9—N2—C11	114.88 (14)	N2—C9—H9B	109.1
C2—C1—C6	118.34 (16)	C10—C9—H9B	109.1
C2—C1—H1	120.8	H9A—C9—H9B	107.9
C6—C1—H1	120.8	C9—C10—H10A	109.5
F1—C2—C3	118.29 (17)	C9—C10—H10B	109.5
F1—C2—C1	118.32 (16)	H10A—C10—H10B	109.5
C3—C2—C1	123.38 (18)	C9—C10—H10C	109.5
C2—C3—C4	117.89 (18)	H10A—C10—H10C	109.5
C2—C3—H3	121.1	H10B—C10—H10C	109.5
C4—C3—H3	121.1	N2—C11—C12	113.38 (15)
C3—C4—C5	121.14 (19)	N2—C11—H11A	108.9
C3—C4—H4	119.4	C12—C11—H11A	108.9
C5—C4—H4	119.4	N2—C11—H11B	108.9
C4—C5—C6	120.0 (2)	C12—C11—H11B	108.9
C4—C5—H5	120.0	H11A—C11—H11B	107.7
C6—C5—H5	120.0	O2—C12—C11	109.28 (13)
C1—C6—C5	119.29 (16)	O2—C12—H12A	109.8
C1—C6—C7	122.51 (15)	C11—C12—H12A	109.8
C5—C6—C7	118.06 (16)	O2—C12—H12B	109.8
O1—C7—N1	123.33 (15)	C11—C12—H12B	109.8
O1—C7—C6	121.38 (15)	H12A—C12—H12B	108.3
N1—C7—C6	115.29 (14)		
			
C6—C1—C2—F1	179.31 (16)	C1—C6—C7—N1	−18.5 (2)
C6—C1—C2—C3	−0.2 (3)	C5—C6—C7—N1	165.87 (16)
F1—C2—C3—C4	−179.56 (19)	C9—N2—C8—N1	170.60 (17)
C1—C2—C3—C4	0.0 (3)	C11—N2—C8—N1	−13.3 (2)
C2—C3—C4—C5	0.0 (3)	C9—N2—C8—S1	−5.4 (3)
C3—C4—C5—C6	0.2 (3)	C11—N2—C8—S1	170.74 (13)
C2—C1—C6—C5	0.5 (2)	C7—N1—C8—N2	139.06 (17)
C2—C1—C6—C7	−175.11 (16)	C7—N1—C8—S1	−44.8 (2)
C4—C5—C6—C1	−0.5 (3)	C8—N2—C9—C10	91.8 (2)
C4—C5—C6—C7	175.31 (19)	C11—N2—C9—C10	−84.6 (2)
C8—N1—C7—O1	−14.3 (3)	C8—N2—C11—C12	93.48 (19)
C8—N1—C7—C6	165.10 (16)	C9—N2—C11—C12	−90.17 (19)
C1—C6—C7—O1	160.93 (17)	N2—C11—C12—O2	−70.51 (19)
C5—C6—C7—O1	−14.7 (2)		

### Hydrogen-bond geometry (Å, º)

**Table:**

*D*—H···*A*	*D*—H	H···*A*	*D*···*A*	*D*—H···*A*
N1—H1*A*···O2	0.86	2.03	2.805 (2)	150
C9—H9*A*···S1	0.97	2.65	3.043 (3)	105
O2—H2*A*···S1^i^	0.82 (3)	2.49 (3)	3.2805 (19)	166 (3)
C11—H11*B*···O1^ii^	0.97	2.44	3.259 (3)	142

Symmetry codes: (i) −*x*, *y*+1/2, −*z*+1/2; (ii) −*x*+1, *y*+1/2, −*z*+1/2.
